# Characteristics of the Mixed Yogurt Fermented from Cow–Soy Milk in the Presence of Transglutaminase

**DOI:** 10.3390/foods13132120

**Published:** 2024-07-03

**Authors:** Xudong Lin, Zhiqi Cao, Jiaxin Zhang, Guangqing Mu, Shujuan Jiang

**Affiliations:** School of Food Science and Technology, Dalian Polytechnic University, Dalian 116034, China; 18242179897@163.com (X.L.); c096zq@163.com (Z.C.); 13604099601@163.com (J.Z.); guangqingmu@163.com (G.M.)

**Keywords:** soy milk, yogurt, texture characteristics, transglutaminase (TG), physicochemical properties

## Abstract

The mixed yogurt was fermented from Cow–Soy milk and modified by transglutaminase (TG). The effects of mixed milk and TG on the quality characteristics of mixed yogurt were investigated by texture characteristics, rheology (rheometer) and structure (scanning electron microscopy). The findings revealed that the mixed yogurt with 50% cow milk exhibited lower hardness, viscosity and consistency. Furthermore, when TG was added, the yogurt showed better rheological properties, sensory score and a more stable microstructure. Compared with the samples without TG modification, the viscosity and cohesiveness of the modified samples increased by 10% and 100%, respectively. The combination of cow milk and soy milk improved the texture of yogurt, and the TG addition further improved the physicochemical properties of yogurt. This finding provided a meaningful reference for the development of mixed yogurt with a suitable taste from animal and plant milk, and laid a basis for the practical application of mixed yogurt in the dairy industry, which will meet the requirements for dairy products for consumers in future.

## 1. Introduction

Soy is a rich source of various nutrients that are beneficial to human health, including protein, lipids, soy isoflavones, vitamins and other ingredients. The content of protein in soy can reach up to 35%, which is significantly higher than that in eggs, beef and pork. Additionally, soy contains essential amino acids. However, soy milk is not well-liked by everyone due to its beany flavor and anti-nutritional factors [1]. Soy milk is obtained by the pre-treatment of soy, and grinding in the appropriate amount of water. Fermentation not only eliminates part of the beany flavor of soy milk and makes it more palatable, but also decomposes the anti-nutritional factors in soy, thereby making the nutritional components more complete and easier to be digested and absorbed by human body [2].

Yogurt is a type of food commonly consumed in daily life which possesses high nutritional value and health benefits. It can help digestion, lower cholesterol, reduce the risk of intestinal diseases and enhance human immunity [3]. ŠERTOVIĆ et al. detected a significant enhancement in mineral content and a notable increase in the concentration of phenolic compounds in the yogurts that were made with a blend of cow milk and soy milk, as opposed to those produced solely from cow milk. Their research indicates that the incorporation of soy milk into cow milk not only enhances the nutritional value but also positively affects the sensory qualities of the fermented dairy products [4]. In a study on fermenting soy milk yogurt and cow milk yogurt with different strains, Li and others found that adding probiotic to yogurt fermented with commercial starter cultures, including the *Bifidobacterium animalis* subsp. *lactis* BB-12 (BB), *L. acidophilus* La-5 (LA), and *L. rhamnosus* (LGG), led to an increased density of the soy milk yogurt’s network and improved the quality of the soy milk yogurt [5]. However, during the post-fermentation phase, soy milk undergoes a biotransformation facilitated by enzymes produced by bacterial organisms, the purpose of which is to augment the flavor profile of soy yogurt. Regrettably, this transformation often leads to the build-up of organic acids, which may negatively impact the stability of the product and alter its texture [6]. TG, also known as protein glutamine γ-glutamyltransferase, is a naturally occurring enzyme that catalyzes the transacylation reaction [7]. It has been approved by the FDA and is considered GRAS (Generally Recognized As Safe). TG can catalyze the cross-linking of glutamine residues in milk protein with lysine to form covalent bonds and generate protein polymers, which can improve some properties, such as the water holding capacity and texture characteristics of proteins and their products [8]. Therefore, it is crucial to apply TG to yogurt products in order to enhance their texture.

Temiz et al. found that the hardness of yogurt samples with the ratio of cow milk to soy milk of 3:1 (*v*/*v*) increased with the increase in TG [9]. However, there are few studies on the interaction of TG and commercial starters on different combinations of soy milk and cow milk. Therefore, in our study, cow milk and soy milk were mixed and fermented under the joint action of TG and commercial starter. The physicochemical properties, sensory characteristics, structure and protein changes of mixed yogurt were studied, aiming to reveal the mixed ratio of soy milk and cow milk and the influence of TG addition on the quality of yogurt. This will provide theoretical support for the development and industrial application of mixed yogurt products from animal milk and vegetable milk with suitable mouthfeel.

## 2. Materials and Methods

### 2.1. Materials

Pasteurized fresh whole fat cow milk was purchased from Xinle Dairy Co., Ltd. (Dalian, Chain). Soy was produced in Liaoning Shenyang and purchased from Xinnong Cereals Co., Ltd. (Shenyang, China). TG with an actual activity of 100 U/g was purchased from Jiangsu Yiming Fine Chemical Industry Co., Ltd. (Qinxing, China), other reagents were analytically pure. YC-X11 starter culture was purchased from Chr Hansen Co., Ltd. (Hirschholm, Denmark); it was freeze-dried culture powder containing *Streptococcus thermophilus* and *Lactobacillus delbrueckii* subsp. *bulgaricus*. The molecular weight label was purchased from Beijing Solarbio Science & Technology Co., Ltd. (Beijing, China)

### 2.2. Sample Preparation

Soy milk was prepared according to the method of a previous study with some modifications [10]. After washing, soy was soaked in six-fold (*w*/*v*) distilled water at 25 °C for 12 h. The water was decanted and the beans were ground in ten-fold (*w*/*v*) of distilled water in a blender. The resulting suspension was filtered through three layers of gauze, and the soy milk was boiled for 20 min to sterilize and remove the beany flavor. After boiling, the soy milk was cooled to room temperature immediately and then kept at 4 °C for storage.

Cow milk and soy milk were mixed in the volume ratio of 75:25 (*v*/*v*), 50:50 (*v*/*v*), and 25:75 (*v*/*v*), which were defined as S25%, S50% and S75%, respectively, to obtain uniform mixed milk. The cow milk (C100%), mixed milk and soy milk (S100%) were heated at 80 °C for 15 min to prepare yogurt samples.

The milk samples (40 mL for each sample) were cooled to room temperature, then inoculated with 0.2 U/L of yogurt starter (*Streptococcus thermophilus* and *Lactobacillus delbrueckii subsp. bulgaricus*). TG was added 0 to 1 U/g protein at the same time, and then cultured at 42 °C. The pH of the yogurt was tested using a pH meter until it reached 4.6, which is the isoelectric point of casein. Yogurt samples were immediately stored at 4 °C for 48 h. All analyses were performed in three independent replicates.

### 2.3. The pH and Titratable Acidity

The pH values of different yogurt samples are measured with a pH meter (FE28, China). The test method for the titration acidity of yogurt originated from the previous paper [11,12].

### 2.4. Apparent Morphology and Microstructure of Yogurt Sample

#### 2.4.1. Chromaticity of Different Yogurt

The chromaticity of different yogurt samples was tested by Konica Minoltacolorimeter CM-700d (Osaka, Japan), according to the previous paper [13].

#### 2.4.2. Water Holding Capacity of Yogurt

The water holding capacity of yogurt was studied by centrifugation according to the previous study [14]. Firstly, the weight of the centrifuge tube was recorded. Then, the yogurt sample was added to the weighed centrifuge tube, and the total weight was recorded again. Next, the sample was centrifuged at 3500 rpm for 5 min, after which the supernatant was poured. The weight of the tube with the remaining yogurt was measured and recorded, then calculated according to the following method:

Water holding capacity = (m_2_ − m_0_)/(m_1_ − m_0_) × 100% (1)

where m_0_ is the weight of empty centrifuge tube, m_1_ is the total weight of the yogurt sample and the centrifuge tube, and m_2_ is the total weight of centrifuge tube and residue after centrifugation.

#### 2.4.3. Scanning Electron Microscope (SEM) of Yogurt

According to the previous research [15], the microstructure of different yogurts was observed by scanning electron microscope (SEM). Small portions of the yogurt samples were taken and fixed with 2.5% (*v*/*v*) glutaraldehyde solution at 4 °C for 12 h. Then, they were soaked and washed with 0.01 mol/L phosphate-buffered solution (PBS) three times. After complete washing, the samples were dehydrated with graded ethanol. Firstly, the yogurt samples were soaked in 30% (*v*/*v*), 50% (*v*/*v*), and 70% (*v*/*v*) ethanol solutions, with each concentration being soaked three times for 10 min each time. Finally, they were soaked with 90% (*v*/*v*) and 100% (*v*/*v*) ethanol, respectively, for 15 min. The eluted yogurt sample was pre-frozen at −80 °C for 12 h, and then freeze-dried. The yogurt sample was observed at an accelerating voltage of 5.0 KV and a magnification of 500×.

### 2.5. Quality Characteristics of Yogurt Sample

#### 2.5.1. Texture Profile Analysis (TPA) of Yogurt

Before determination, the height was calibrated; the calibration height was set at 60 mm. The probe was configured to penetrate into the sample for 10 mm, and the probe type P/0.5 was selected. The test speed was set to 5 mm/s. According to the method described by previous paper [16], some modifications were made and the texture (TPA) analysis was carried out. The yogurt samples were placed on the TA-XT Plus texture analyzer (Stable Micro systems, Surrey, UK), and the corresponding test program was selected for TPA determination. Before determination, the height was calibrated, the calibration height was set at 60 cm. The probe was configured to penetrate into the sample for 10 mm and the probe was selected for P/0.5. The test speed was set to 5 mm/s.

#### 2.5.2. Rheological Properties of Yogurt

The rheological properties of yogurt sample were assessed according to the previous method [17]. Some modifications were made, and the corresponding test program was selected on the rotary rheometer (KNX2212, Malvern Instruments Limited, Worcestershire, UK) according to the following conditions. Before the experiment, the yogurt samples were stored in the refrigerator at 4 °C. During the experiment, the samples were taken out, left for 2 h to reach room temperature (25 °C), and the appropriate amount of yogurt samples were loaded on the parallel plate of the rheometer. The fixture of the rotary rheometer used in the experiment was a parallel plate with a diameter of 40 mm, and the gap between the testing tool and the sample testing table was 1 mm and the testing temperature was 25 °C. The samples were tested in the shear rate range of 1–1000 s^−1^, and the change in shear viscosity (η) with the shear rate was monitored. In the linear viscoelastic region, the shear strain was 1%, and storage modulus (G′) and loss modulus (G″) were measured at 0.1–10 Hz with the change in frequency.

#### 2.5.3. Flavor of Yogurt

The analysis of flavor was performed according to Yang et al. with some modifications [18]. The flavor was tested by portable electronic nose (PEN3 AIRSENSE, Schwerin, Germany) with ten metal oxide sensors. An automatic headspace sampling system was used to detect the changes in the aroma at room temperature. The names and performance descriptions of the sensors of the electronic nose are shown in Table 1. To quantify the overall color difference, ΔE was calculated using the provided formula (2):

ΔE = [(ΔL*)^2^ + (Δa*)^2^ + (Δb*)^2^]^1/2^ (2)

#### 2.5.4. Sensory Analysis of Yogurt

Sensory analysis was performed according to the previous study with some modifications [19]. Firstly, a sensory evaluation team composed of ten well-trained members tested the samples, including the color, flavor and organizational structure of the samples. After 48 h of post-ripening, an appropriate amount of yogurt sample was taken and put in a tasting cup, randomly numbered and provided to the tasters. Each appraiser scored the randomly provided yogurt samples and gargled with purified water after each evaluation. During the evaluation process, each appraiser evaluated independently without any connection. The testing results of the yogurt samples were collected and sorted by the appraisers. The scoring criteria are as follows:

The highest score of a single item cannot exceed the score specified in a single item. The total score was the sum of individual scores of each evaluator, which was used for the overall evaluation of the product, and the individual score was used for the individual evaluation of the mixed yogurt sample. The total score is calculated according to the following formula. Excluding the highest and lowest scores in the total score, the result is an integer:

Total score = Sum of other total scores/(evaluation number − 2)(3)

### 2.6. Protein Structural Changes in Yogurt Sample

An analysis of protein molecular weight was performed according to the previous study with some modifications [20]. The yogurt samples were mixed with the sample buffer containing β-mercaptoethanol at a ratio of 1:1 (*v*/*v*) and boiled for 5 min. A total of 15 μL sample solution was added to the prepared gel (including 5% stacking gel and 15% acrylamide separating gel), and the experiment was carried out under the condition of a constant voltage of 80 v. When the protein bands reach the interface between the concentrate gel and the separating gel, the voltage was increased to 120 V, sustained until the protein bands reached the bottom of the gel, and the experiment ended. The dye containing 0.1% Coomassie Brilliant Blue R250 and 6.8% (*v*/*v*) acetic acid was dissolved in 50% methanol for dyeing on the sample gel. Then, a decolorizing solution containing 50% (*v*/*v*) methanol and 7.5% (*v*/*v*) acetic acid was used to decolorize the gel.

### 2.7. Statistical Analysis

Data were presented as the mean ± standard deviation. Statistical analyses were performed by the SPSS statistical software (SPSS 18.0 SPSS Inc., Chicago, IL, USA) to conduct ANOVA and Duncan tests to analyze the differences between the test values.

## 3. Results and Discussion

### 3.1. The pH Change in the Yogurt Sample during Fermentation and Titration Acidity after Post-Fermentation for 48 h

#### 3.1.1. pH Analysis

**Figure 1:**
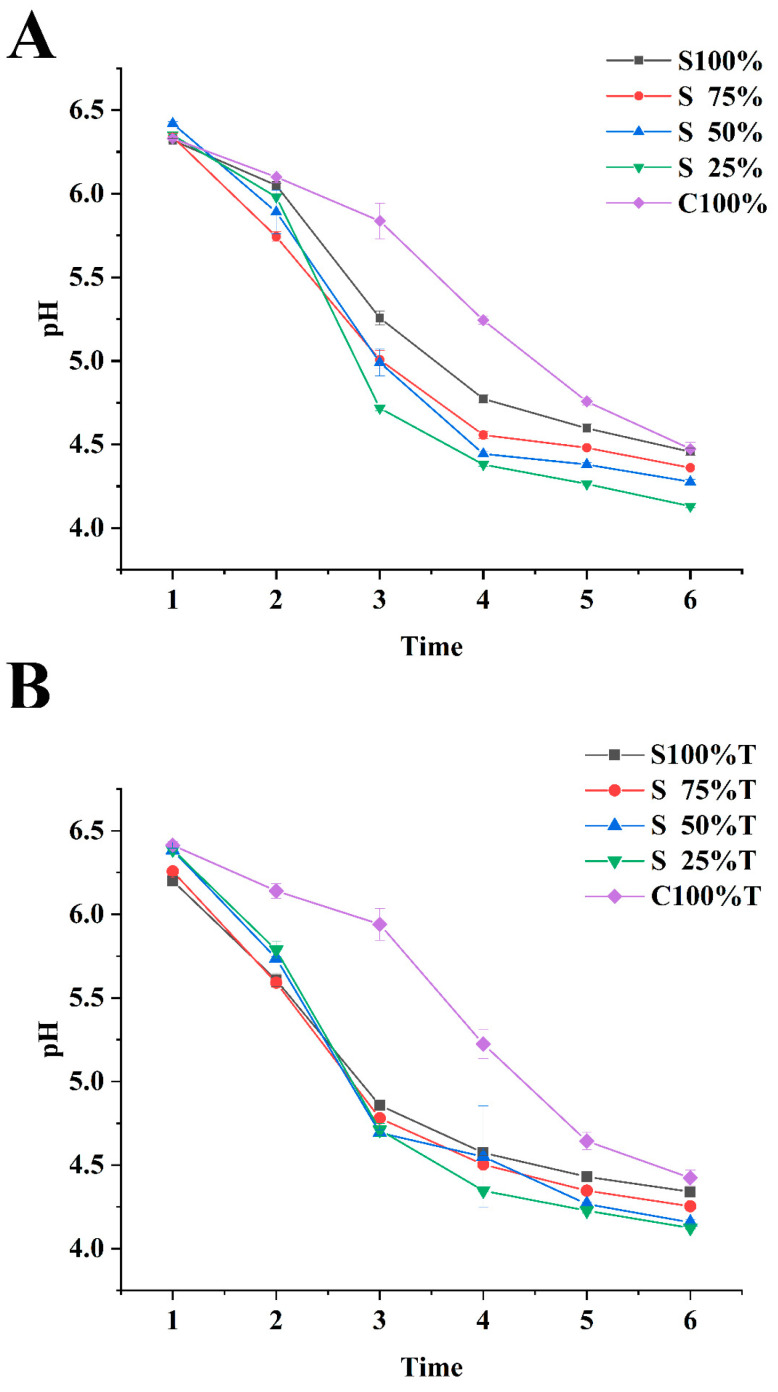
pH of sample during fermentation. (**A**): sample without TG, (**B**): sample with TG. Values are given as the mean ± standard deviation. Note: T: transglutaminase, S100%, S75%, S50%, S25%, C100% defines the yogurt fermented from 100% soy milk, 75% soy milk, 50% soy milk, 25% soy milk and 100% cow milk, respectively. These same definitions will not repeatedly described again in other Figures.

Figure 1 illustrated the pH changes in yogurt samples, which were a result of microbial activity during the fermentation process. It was evident from the graph that as fermentation duration increases, the pH of all samples uniformly trends downward. Additionally, the pH of mixed yogurt (S75%, S50%, S25%) decreased faster than that of yogurt prepared by soy milk and cow milk alone, which was consistent with the findings of previous studies [21,22]. This was ascribed to the presence of antinutritional factors in soybeans that hinder the growth of strains involved in fermentation [23]. The yogurt made entirely from soy milk did not provide a sufficient nutritional environment for the fermenting strains to grow in during the fermentation process. The slow decrease in pH, observed in the yogurt made using 100% cow milk as the raw material, might have been caused by the higher levels of buffering substances present in cow milk. These substances could have neutralized the lactic acid that was formed during the fermentation process. By contrast, the yogurt made from a blend of different raw materials contained nutrients that facilitated the usual growth of the fermenting strains and had an adequate buffering capacity, leading to a more substantial decrease in pH than that observed in yogurt fermented with a single material. Shahabbaspour et al. studied the effects of different proportions of cow milk and soy milk, different probiotics (*Lactobacillus acidophilus LA-5* or *Lactobacillus casei L-01*) and natural concentrated fruits on the quality characteristics of soy probiotic drinks [21]. They found that the pH of yogurt fermented with 75:25 (*v*/*v*), 50:50 (*v*/*v*) and 25:75 (*v*/*v*) cow milk powder and soy milk decreased rapidly, while the pH of yogurt fermented with 100:0 and 0:100 cow milk powder and soy milk decreased slowly. The pH of mixed yogurt samples, with added TG and prepared from raw materials with a high content of soy milk, decreased rapidly within 2–3 h, and the acidification rate of yogurt samples prepared from 100% soy milk, especially, increased significantly, as shown in Figure 1B. Compared to the samples without the addition of TG, the yogurt sample that was supplemented with TG and made from 100% cow milk showed a faster acidification rate during the initial 3–5 h. After 5 h of fermentation or more, the pH of yogurt samples with and without TG became similar.

#### 3.1.2. Titration Acidity Analysis

**Figure 2:**
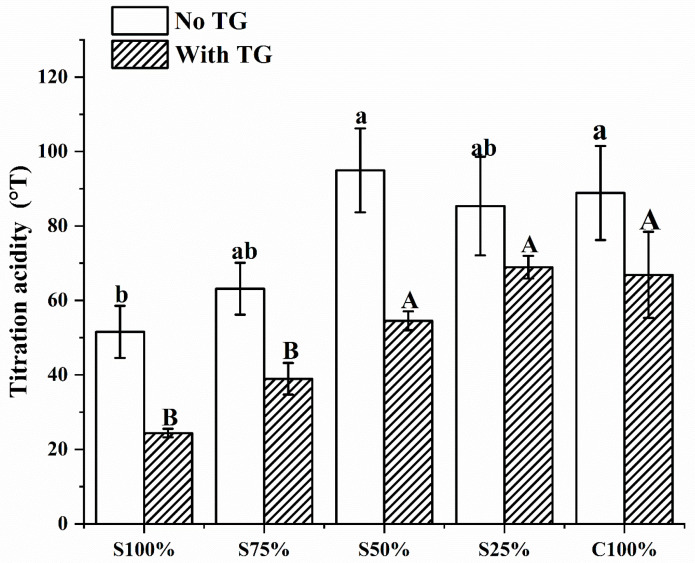
Titration acidity of yogurt sample. Different lowercase letters mean significant differences between samples without TG (*p* < 0.05). Different capital letters mean significant differences between samples with TG (*p* < 0.05). Values are given as the mean ± standard deviation.

The titration acidity of the yogurt samples is shown in Figure 2. When the proportion of cow milk was below 50%, the titration acidity of the yogurt samples increased as the proportion of cow milk rose. When it was at 50%, the titratable acidity reached its peak, and there was no significant alteration in titratable acidity as the proportion of cow milk continued to increase. Due to cow milk’s strong buffering capacity, the proportion of cow milk in the mixed yogurt was lower than that in pure cow milk fermented yogurt, resulting in a reduced buffering capacity. Thus, when the proportion of cow milk was below 50%, the titratable acidity of mixed yogurt was substantially lower than that of the yogurt fermented solely from cow milk. Meanwhile, the titratable acidity of yogurt fermented without cow milk, namely soy milk fermented yogurt, was the lowest [21]. In addition, the acidity of yogurt samples with TG showed a trend of acidity change consistent with samples without TG, but the overall acidity was significantly lower than that of samples without TG. The addition of TG mitigated the rise in acidity, an effect attributed to its inhibition of microbial proliferation [24]. In conclusion, in the mixed fermentation system of soy milk and cow milk, the increase in the proportion of soy milk and the addition of TG were found to reduce titratable acidity.

### 3.2. Apparent Characteristics of Mixed Yogurt

#### 3.2.1. Chromaticity Analysis

When purchasing yogurt, the most straightforward criterion for consumers is often the yogurt’s color. The color data were represented by L*, a* and b*, which represent the measured values of the samples and can be directly read from the chromaticity value meter. L* stood for brightness, and the greater the L* value of the sample, the higher the brightness of the sample. a* stood for red–green values, and + a* indicates that the color tends to turn red, and − a* indicates that the color tends to turn green. b* stands for yellow–blue values, + b* indicates that the color tends to yellow, and − b* indicates that the color tends to blue.

The chromaticity values of yogurt samples are shown in Table 2 and Table 3. It can be seen that the L* value of yogurt samples increases with the increase in cow milk proportion. In all mixed yogurt samples, S100% and C100% a* values showed significant differences (*p* < 0.05), which means that yogurt fermented with different proportions of soy milk and cow milk has different red and green values. In Table 2, it was found that the addition of TG improved the brightness of yogurt samples. This effect may be attributed to TG, which can promote the cross-linking of proteins in yogurt, leading to a denser and smoother structure. Therefore, the reflectivity of light on the surface of yogurt was improved, thus improving the overall brightness of yogurt. This phenomenon aligns with the findings of Feng et al.‘s research on the effect of transglutaminase on the gel properties of surimi and early maturing Chinese mitten crab meat [25], where the addition of TG was shown to increase the protein gel density, thereby reflecting more light and brightening the sample.

#### 3.2.2. Water Holding Capacity (WHC) Analysis

**Figure 3:**
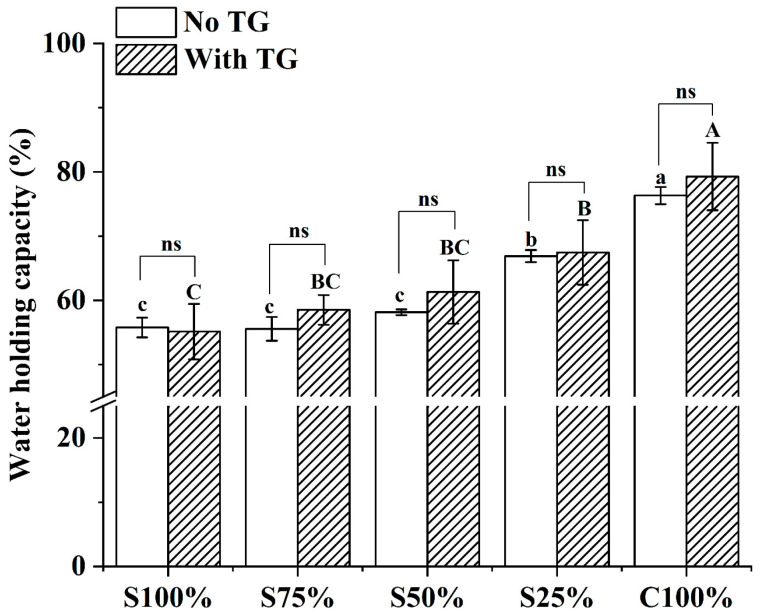
Water holding capacity of yogurt sample. Different lowercase letters mean significant differences between samples without TG (*p* < 0.05). Different capital letters mean significant differences between samples with TG (*p* < 0.05). ns: there is no significant difference. Values are given as the mean ± standard deviation.

Yogurt is a solid–liquid mixed gel system. WHC is animportant quality characteristics of yogurt samples, which reflects the water-fixing ability of the solid substrate of yogurt [8]. As depicted in Figure 3, the WHC of all yogurt samples also increased with the increasing proportion of cow milk in raw materials. The increase in cow milk reduced the contraction of the yogurt gel, thus improving its WHC [26]. TG can catalyze the lysine residue of protein in yogurt samples to form a stable three-dimensional network structure, so that its gel network can retain more water, thus improving the water holding capacity of yogurt. [27]. In Figure 3, compared to the yogurt sample without TG, the WHC of the yogurt sample treated by TG increased slightly, but there was no significant difference. Therefore, the WHC of yogurt can be increased by using more cow milk in the raw materials and by adding TG, which enhances the stability of the yogurt’s gel network and its WHC.

#### 3.2.3. Microstructure Analysis

**Figure 4:**
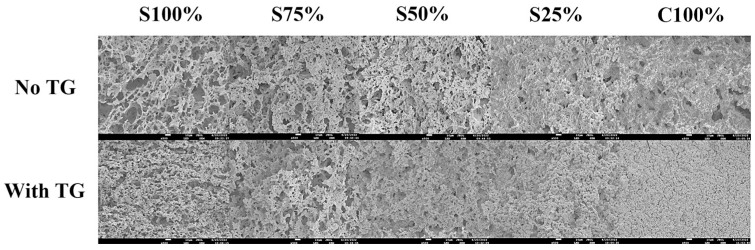
SEM observation of yogurt sample. (Set yogurt sample with 500 times magnification).

Figure 4 illustrates the microstructures (magnified 500×) of yogurt samples prepared from cow milk and soy milk in varying ratios. It is evident that all samples exhibited a honeycomb-like structure, although there were noticeable variations in the distribution of the network structure and pore size. As shown in Figure 4, as the proportion of cow milk increased, the yogurt samples became progressively denser with fewer holes and a more uniform microstructure. This trend was also observed in samples treated with TG. The data suggest that the addition of soy milk weakened the gel matrix connectivity, resulting in the formation of more numerous and larger pores. A parallel finding was reported by Han et al. in their investigation of the impact of TG on the gelation of the skim milk and soy milk mixture induced by rennet [28]. Yogurt samples processed with TG exhibited a denser and more consistent structure compared to those without TG treatment. Additionally, Tsevdou et al. examined the quality of solidified yogurt by treating milk with high pressure (HP) and transglutaminase (TG) (alone or in combination) [29]. They observed that TG-treated yogurt samples exhibited a more compact gel structure.

### 3.3. Quality Characteristics of Yogurt Samples

#### 3.3.1. TPA Analysis

**Figure 5:**
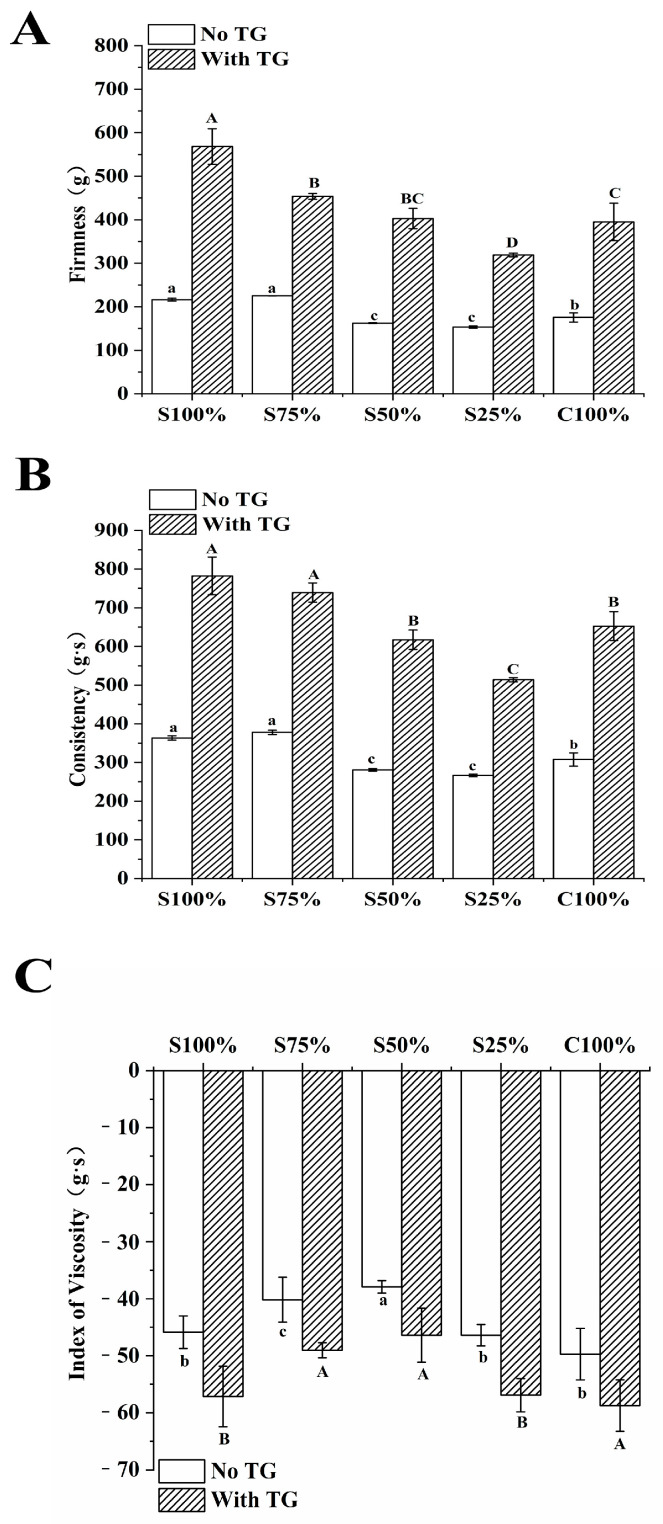
Firmness (**A**), Consistency (**B**) and Index of Viscosity (**C**) of yogurt sample. Different lowercase letters mean significant differences between samples without TG (*p* < 0.05). Different capital letters mean significant differences between samples with TG (*p* < 0.05). Values are given as the mean ± standard deviation.

The TPA parameter serves as a critical indicator for yogurt quality assessment. When assessing yogurt’s texture, factors such as hardness, consistency, and viscosity are commonly evaluated. These properties were determined by subjecting yogurt samples to a continuous application of force using a test probe. The obtained test results were presented in Figure 5. Figure 5A,B clearly demonstrated that the hardness and consistency of yogurt samples exhibit an initial decrease and then an increase with the increase in cow milk content in the ingredients. These properties reached their minimum when the cow milk proportion was 75%.

Regarding the Index of Viscosity, as depicted in Figure 5C, the yogurt samples’ viscosity initially decreases and then increases as the cow’s milk content increases. The mixed yogurt sample containing 50% soy milk had low hardness and viscosity. The research by Kailasapathy et al. showed that when people chewed high-viscosity yogurt, great viscosity will be produced in the mouth, which will have a negative impact on the taste and texture of yogurt [30]. Compared with the yogurt samples without TG, the yogurt samples with TG had a similar trend, with their texture characteristics significantly improved (*p* < 0.5). The results showed that TG catalyzed the protein cross-linking between soy milk and cow milk, formed high molecular weight protein polymers and a denser gel structure, which increased the yogurt samples hardness, consistency and viscosity [31].

#### 3.3.2. Rheology Analysis

**Figure 6:**
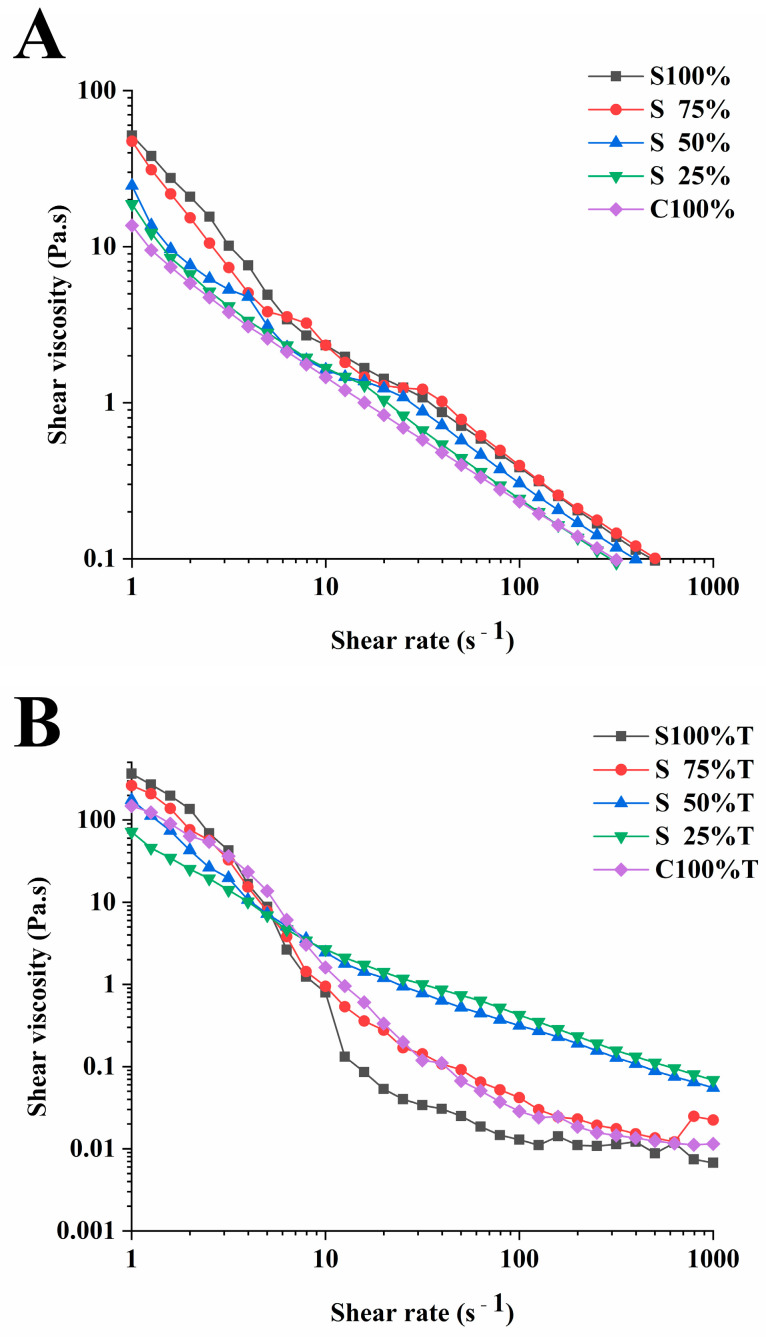
Apparent viscosity changes of yogurt sample under steady-state shear scanning. (**A**): yogurt sample without TG, (**B**): yogurt sample with TG.

The apparent viscosity is an important index affecting the quality of yogurt and can reflect the stability of samples. Figure 6 illustrates the change in apparent viscosity of yogurt samples. Figure 6A demonstrates that as the soy milk content in the yogurt increased, so did the yogurt’s viscosity. Previous research by Mitra et al. had shown that the inclusion of soy protein resulted in increased viscosity within yogurt [32]. The addition of TG significantly enhanced the yogurt’s apparent viscosity (*p* < 0.05), as depicted in Figure 6B. Similar findings were reported by Yang et al., who observed that TG can improve the viscosity of gluten in the study of applying transglutaminase (TGase) and rice protein isolate (RPI) to the production of rice noodles [33]. At higher shear rates, yogurt samples containing 50% soy milk exhibited elevated shear viscosity. It can be observed from Figure 6 that the apparent viscosity of all samples gradually decreases with the increase in shear rate, which is a typical shear thinning behavior of non-Newtonian fluids [34]. Presumably, this was because, with the increase in shear rate, the aggregated protein network structure in yogurt samples were destroyed, which lead to the decrease in shear resistance and shear viscosity.

**Figure 7:**
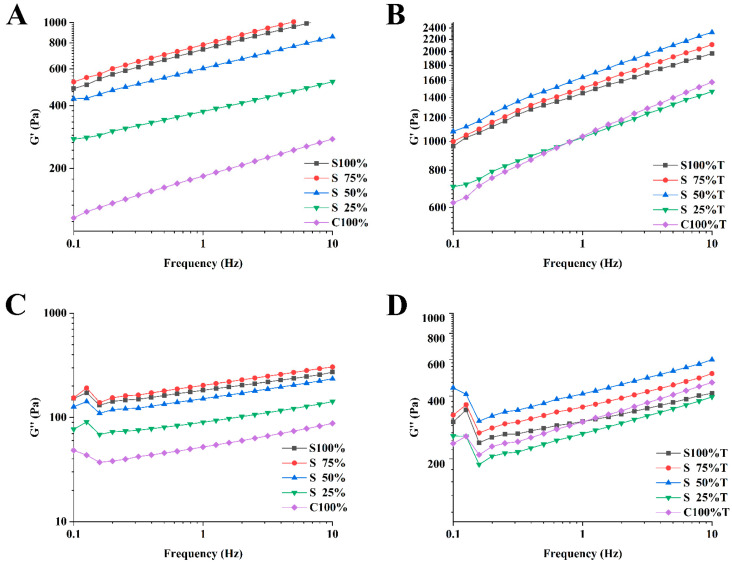
Frequency scanning of yogurt sample with elastic modulus (**A**,**B**) and viscous modulus (**C**,**D**). (**A**,**C**): yogurt sample without TG, (**B**,**D**): yogurt sample with TG3.3.3. Flavor analysis.

G′ and G″ represent the elastic and viscous characteristics of yogurt samples [35]. Figure 7 illustrates that both the elastic modulus and the viscous modulus generally rose with frequency for all yogurt samples, with the elastic modulus consistently surpassing the viscous modulus. This indicated that elastic behavior predominates in yogurt samples, which were predominantly in a solid state. Among the samples tested, those containing 75% soy milk exhibited the highest viscoelasticity, followed by those with 100% soy milk, with other samples showing a decrease as cow milk content increased, as depicted in Figure 7A,C. Figure 7B,D reveal that the mixed yogurt samples containing 50% soy milk had the highest viscoelasticity due to the addition of TG. The addition of TG significantly enhanced the viscoelasticity of all samples compared to the samples without TG. This effect may be attributed to the fact that TG can catalyze the cross-linking of protein in cow milk and soy milk, thus forming a denser protein network and improving the viscoelasticity of yogurt samples. Li et al. also had similar findings in their study [36].

**Figure 8:**
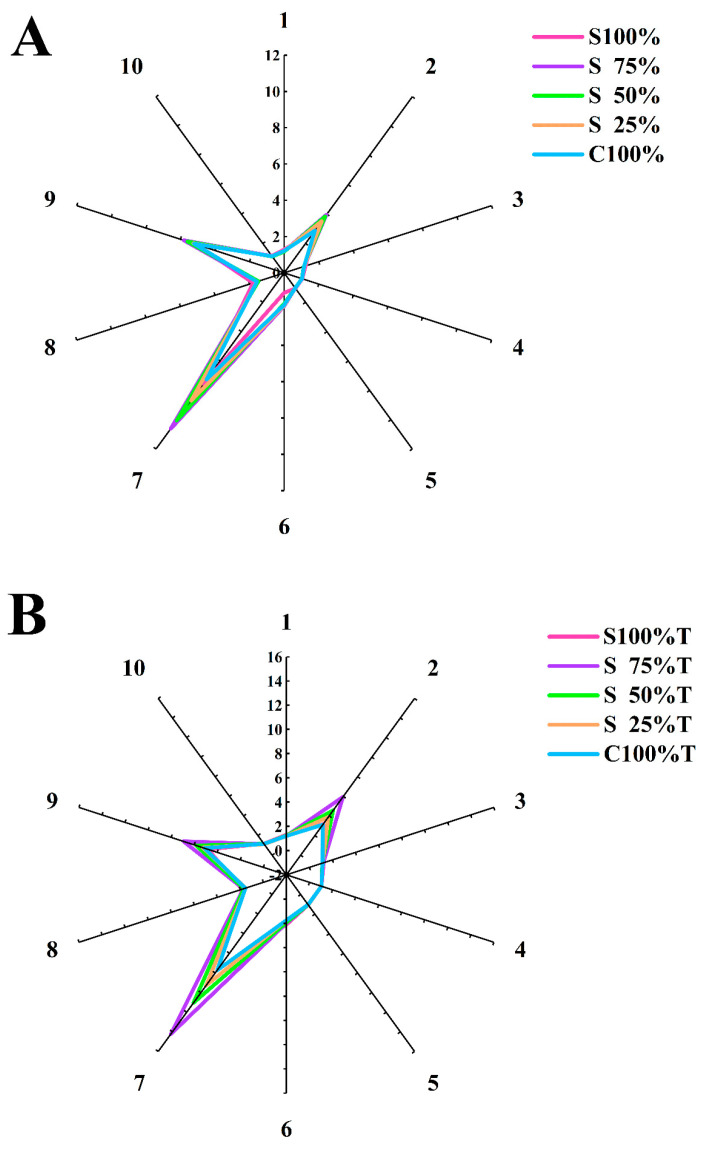
Flavor analysis of yogurt. (**A**): yogurt sample without TG, (**B**): yogurt sample with TG.

The results of yogurt flavor evaluation by sensors are shown in Figure 8. The radar chart can clearly reflect the signal strength of ten sensors of the electronic nose. As depicted in Figure 8, yogurt samples yielded strong signals for sensors No. 2, No. 7, and No. 9. Table 1 revealed that sensor No. 2 was highly responsive to nitrogen oxides, sensor No.7 to sulfides, and sensor No. 9 to both aromatic compounds and organic sulfides. This indicated that aromatic compounds significantly contribute to the aroma detection in mixed yogurt. After TG treatment, the lines in Figure 8A became distinctly separable, suggesting that TG increased the flavor substance content in yogurt samples and generated more volatile components, including W5S (nitrogen oxides), W1W (sulfide), and W2W (organic sulfide). This was attributed to the fact that TG catalyzed the cross-linking of soy and cow milk protein, exposing additional disulfide bonds and sulfhydryl groups. This led to the yogurt samples treated with TG showing stronger signals on sensors No. 2 and No. 9 [37]. Figure 8B shows that as the soy milk content increased, the signals from sensors No. 2, No. 7 and No. 9 for the mixed yogurt samples progressively intensified. Furthermore, the yogurt samples made from 100% cow milk exhibited lower electrical signals, and this trend was also observed in the TG-treated mixed yogurt sample (Figure 8A).

#### 3.3.3. Sensory Analysis

The sensory evaluation of yogurt is presented in Table 4 and Table 5. The sensory properties of yogurt samples added with TG have been improved, and it was more popular with appraiser. It was observed that the texture and total score of yogurt added with TG were significantly improved, especially in yogurt samples fermented with 100% soy milk. Concurrently, as the proportion of cow milk increased, the yogurt’s score also ascended. This was attributed to the fact that mixed yogurt fermented with soy milk had a beany smell and an obvious yellow color. Therefore, with the increase in cow milk proportion and the addition of TG, yogurt samples were more popular.

### 3.4. Change in Milk Protein Structure in Yogurt Sample

**Figure 9:**
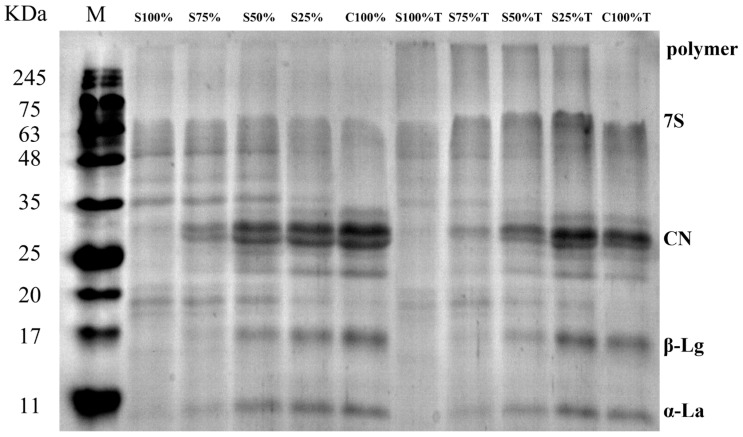
SDS-PAGE profiles of yogurt sample. M(Marker)Lanes for protein marker Lane.

As shown in Figure 9, SDS-PAGE was used to explain the cross-linking occurrence between yogurt proteins catalyzed by TG. Lane M was the standard for protein molecular weights, with protein molecule movement in the electrophoresis map being primarily influenced by their relative molecular weights. Figure 9 reveals that the protein content in yogurt samples, particularly in the 25–35 kDa range, increased as the cow milk content increased, a trend also observed in the samples treated with TG. Comparing the yogurt samples in Lane S100% to C100% and Lane S100%T to C100%T, it was evident that the addition of TG resulted in a reduction in the casein and whey protein bands. Additionally, there was an accumulation of protein polymers at the top in Lane S100%T to C100%T, a phenomenon not observed in samples without TG treatment [38]. This might be the cross-linking by TG between soy protein and cow milk protein, which led to the aggregation of small molecular proteins and the formation of macromolecular polymers, and thus caused the structural changes to the proteins.

## 4. Conclusions

In this work, soy milk and cow milk were co-fermented in the presence of TG. Regardless of whether TG was added or not, compared with pure soy milk yogurt and pure milk yogurt samples, the yogurt samples containing a 50% soy milk fraction exhibited reduced viscosity, hardness, and consistency. The mixed yogurt also had good rheological properties and a relatively compact microstructure. Moreover, the addition of TG elevated the yogurt’s viscosity and hardness, improved sensory attributes, and provided a smoother, more stable texture. The content of sulfide and aromatic compounds in the yogurt samples with TG addition was also higher. This study showed that the addition of TG can improve the physicochemical properties of mixed yogurt, which will provide theoretical support for the development and industrial application of mixed yogurt from animal milk and vegetable milk with suitable mouthfeel.

## Figures and Tables

**Table 1:** Description of the sensors and corresponding aroma types of electronic nose.

Serial Number	Sensor Name	Performance Description
1	W1C	Aromatic components (benzene)
2	W5S	High sensitivity, sensitive to nitrogen oxides
3	W3C	Ammonia, sensitive to aromatic components
4	W6S	Mainly selective for hydrogen
5	W5C	Alkane aromatic component
6	W1S	Sensitive to methyl groups
7	W1W	Sensitive to sulfide
8	W2S	Sensitive to alcohols and aldehydes and ketones
9	W2W	Aromatic component, sensitive to organic sulfide
10	W3S	Sensitive to alkanes

**Table 2:** Chromaticity value of yogurt with TG.

Sample	L*	a*	b*	ΔE
S100%T	108.33 ± 0.06 ^d^	−157.50 ± 0.00 ^a^	19.21 ± 0.25 ^a^	25.11 ± 5.71 ^b^
S75%T	117.83 ± 0.59 ^c^	−169.80 ± 0.6 ^b^	20.14 ± 0.24 ^a^	42.21 ± 0.23 ^a^
S50%T	118.77 ± 0.29 ^c^	−169.67 ± 0.74 ^b^	19.30 ± 0.34 ^a^	44.08 ± 0.38 ^a^
S25%T	120.93 ± 0.15 ^b^	−172.40 ± 0.62 ^bc^	19.42 ± 0.70 ^a^	46.39 ± 0.81 ^a^
C100%T	122.87 ± 1.61 ^a^	−173.40 ± 3.95 ^d^	16.89 ± 3.70 ^a^	48.41 ± 4.77 ^a^

Values are given as the mean ± standard deviation. Lowercase letters mean significant differences between samples with TG (*p* < 0.05).

**Table 3:** Chromaticity value of yogurt without TG.

Sample	L*	a*	b*	ΔE
S 100%	93.22 ± 0.20 ^e^	−137.23 ± 0.47 ^a^	21.42 ± 0.19 ^a^	6.28 ± 0.64 ^d^
S75%	115.10 ± 0.00 ^d^	−165.50 ± 0.10 ^b^	19.28 ± 0.15 ^b^	37.72 ± 0.10 ^c^
S50%	116.60 ± 0.10 ^c^	−167.17 ± 0.12 ^c^	19.01 ± 0.12 ^b^	40.29 ± 0.12 ^b^
S25%	118.40 ± 0.10 ^b^	−167.07 ± 0.15 ^c^	15.07 ± 0.24 ^c^	40.54 ± 0.17 ^b^
C100%	120.37 ± 0.31 ^a^	−167.83 ± 0.80 ^c^	12.44 ± 0.34 ^d^	41.58 ± 0.72 ^a^

Values are given as the mean ± standard deviation. Lowercase letters mean significant differences between samples without TG (*p* < 0.05). Note: T: transglutaminase, S100%, S75%, S50%, S25%, C100% defines the yogurt fermented from 100% soy milk, 75% soy milk, 50% soy milk, 25% soy milk and 100% cow milk, respectively. These same definitions will not be repeatedly described again in other Tables.

**Table 4:** Sensory attributes of yogurt with TG.

Sample	Color	Flavor and Odor	Texture	Total Score
S100%T	7.67 ± 0.58 ^c^	12.00 ± 1.00 ^c^	15.00 ± 1.00 ^c^	35.33 ± 0.58 ^d^
S75%T	8.33 ± 0.58 ^bc^	10.67 ± 1.15 ^bc^	15.67 ± 0.58 ^c^	36.33 ± 0.58 ^d^
S50%T	8.67 ± 0.58 ^b^	13.00 ± 1.00 ^b^	17.00 ± 0.00 ^b^	37.67 ± 0.58 ^c^
S25%T	9.00 ± 0.00 ^b^	16.67 ± 0.58 ^a^	18.00 ± 0.00 ^ab^	39.67 ± 0.58 ^b^
C100%T	10.00 ± 0.00 ^a^	17.67 ± 0.58 ^a^	18.33 ± 0.58 ^a^	41.00 ± 1.00 ^a^

Values are given as the mean ± standard deviation. Lowercase letters mean significant differences between samples with TG (*p* < 0.05).

**Table 5:** Sensory attributes of yogurt without TG.

Sample	Color	Flavor and Odor	Texture	Total Score
S 100%	6.33 ± 0.58 ^d^	9.00 ± 1.00 ^b^	8.33 ± 0.58 ^d^	24.00 ± 0.00 ^e^
S75%	7.33 ± 0.58 ^cd^	11.00 ± 1.73 ^b^	12.00 ± 1.00 ^c^	27.33 ± 1.53 ^d^
S50%	8.33 ± 0.58 ^bc^	18.00 ± 1.00 ^a^	14.67 ± 0.58 ^b^	35.00 ± 0.00 ^c^
S25%	8.67 ± 0.58 ^ab^	17.00 ± 1.73 ^a^	14.33 ± 0.58 ^b^	37.00 ± 1.00 ^b^
C100%	9.67 ± 0.58 ^a^	17.67 ± 1.53 ^a^	17.67 ± 1.53 ^a^	41.33 ± 0.58 ^a^

Values are given as the mean ± standard deviation. Lowercase letters mean significant differences between samples without TG (*p* < 0.05).

## Data Availability

The original contributions presented in the study are included in the article, further inquiries can be directed to the corresponding author.
